# Diagnostic Challenges in Adult Intestinal Malrotation: A Case Report and Literature Review

**DOI:** 10.7759/cureus.52281

**Published:** 2024-01-15

**Authors:** Chirwa Abdillahi Mahamoud, Abdoulrazak Egueh Nour, Nawal Bouknani, Najwa Benslima, Amal Rami

**Affiliations:** 1 Department of Radiology, Cheikh Khalifa International University Hospital, Mohammed VI University of Health Sciences, Casablanca, MAR

**Keywords:** pancreas abnormalities, small abnormalities, intestine, computed tomography, congenital abnormalities, adult intestinal malrotation

## Abstract

Adult intestinal malrotation is a rare anatomical anomaly that typically manifests during infancy but can also present in adulthood. Symptoms are mainly digestive, with a long history of intermittent abdominal pain and epigastralgia. It often presents a diagnostic challenge due to the varied and nonspecific nature of clinical symptoms. Radiological evaluations reveal diverse patterns of malrotation, including incomplete rotation, mesenteric base abnormalities, and associated malformations. Computed tomography (CT) scans consistently identify characteristic anatomical distortions, aiding in accurate diagnosis.

In this context, we present a unique case in which contrast-enhanced CT of the abdomen, initially conducted to investigate a suspected episode of acute pancreatitis accompanied by epigastralgia, fortuitously revealed the presence of intestinal malrotation. Once the diagnosis has been made, the therapeutic approach is based on monitoring or managing complications such as intestinal obstruction.

Early recognition and accurate radiological assessment of intestinal malrotation play an essential role in establishing the diagnosis and guiding appropriate management strategies. Increased awareness among clinicians and radiologists is essential to avoid delays in diagnosis and the potential complications associated with this entity.

## Introduction

Intestinal malrotation is a congenital developmental anomaly of the digestive tract during fetal life. During the complex process of its development, errors in the rotation of the umbilical loop can sometimes cause serious complications [1]. With an incidence of 1 per 6,000 births, this anomaly is diagnosed during the first year of life in 90% of cases [2]. Extremely rare in adults, with an incidence of between 0.00001% and 0.19% [1], most intestinal malrotations are diagnosed incidentally during surgery or imaging [3,4].

In adults, symptoms are principally characterized by sporadic abdominal pain, epigastralgia, biliopancreatic manifestations, and vomiting, although a subset of patients remains asymptomatic [4]. The correlation with congenital anomalies, especially those pertaining to the pancreas, is typically fortuitously identified [5,6]. The gravest complication pertains to the occurrence of complete small bowel volvulus, a phenomenon observed in approximately 1% to 2% of the instances delineated within pediatric cases [7].

We report a rare case of a 32-year-old male patient presenting with acute, transfixing abdominal pain radiating to the back who was incidentally found by computed tomography (CT) to have intestinal malrotation with aplasia of the uncinate process of the pancreas.

## Case presentation

A 32-year-old male patient sought consultation following an episode of acute, transfixing abdominal pain, accompanied by radiating discomfort to the back, epigastralgia, and concurrent nausea. Notably, the patient also presented with respiratory symptoms characterized by a productive cough that yielded greenish sputum. Pertinent aspects of the patient’s social history included ongoing tobacco use with a cumulative exposure of 10 pack-years, sporadic alcohol consumption, and persistent occupational stress. Significantly, there was an absence of any prior surgical interventions or documented drug allergies in the patient’s medical history.

The patient remained hemodynamically stable, with a blood pressure of 115/68 mmHg, a pulse rate of 72 beats per minute, and an oxygen saturation of 98% while breathing room air. Abdominal examination revealed no notable abnormalities. Auscultation unveiled a few deviations related to the symptoms of cough and secretions, while his respiratory status remained unchanged. The prominent presenting symptom involved acute, intense pancreatic pain accompanied by epigastralgia, which had manifested over 48 hours.

**Figure 1:**
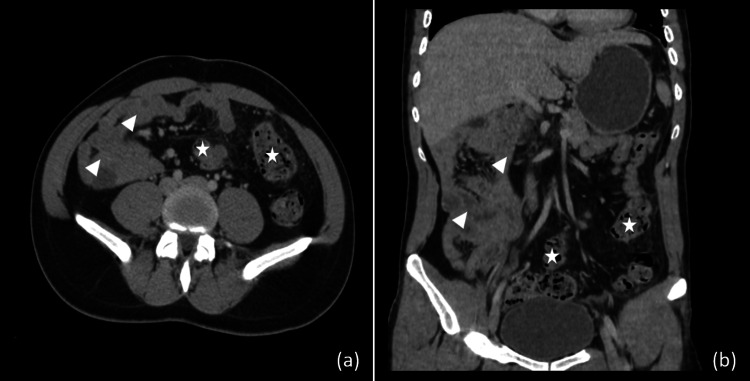
The CT scan shows, in axial (a) and coronal (b) sections, the colon on the left side of the abdomen (white star), and the small intestine on the right side (arrowhead).

Following these assessments, an abdominal-pelvic CT scan, performed with contrast injection, was conducted to detect signs of acute pancreatitis. The findings disclosed a configuration in which the entire length of the small bowel, inclusive of the duodenojejunal juncture, assumed a position to the right of the spinal column, while the entire colon aligned to the left (Figures 1a, 1b).

**Figure 2:**
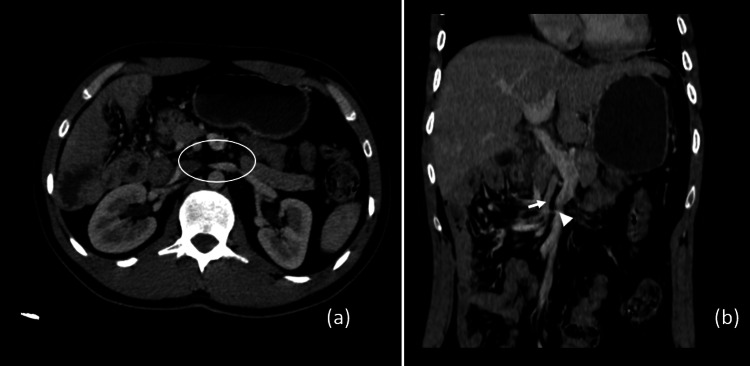
CT images demonstrating (a) the absence of the third portion of the duodenum in the aorto-mesenteric clamp and (b) the abnormal superior mesenteric artery-superior mesenteric vein (SMA-SMV) relationship associated with intestinal malrotation, with the SMV (arrowhead) being to the left of the SMA (full arrow).

Notably, there was an absence of a third portion of the duodenum within the aorto-mesenteric clamp setup (Figure 2a). Additionally, there was an inversion in the customary anatomical relationship of the superior mesenteric vessels, as evidenced by the vein traversing to the left of the artery (Figure 2b).

**Figure 3:**
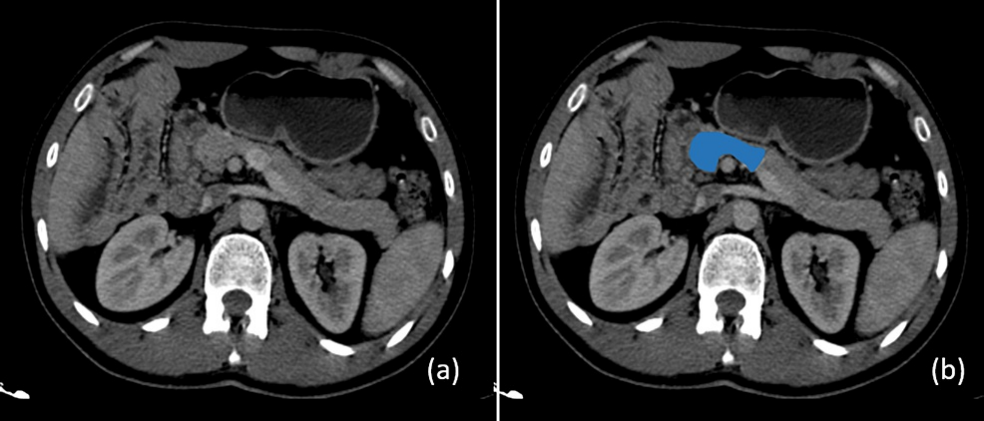
Axial section (a) of an aplastic uncinate process. No pancreatic tissue is visible behind the superior mesenteric vein. (b) For clarification, the pancreas with its globular head is marked in blue, highlighting the aplasia of the uncinate process.

Coincidentally, a diagnosis of uncinate process aplasia of the pancreas was ascertained; this condition exhibits no irregularities concerning the pancreatic ducts (Figures 3a, 3b).

The ultimate diagnosis remained indicative of a complete common mesentery, concomitant with uncinate process aplasia of the pancreas, albeit devoid of any involvement of pancreatic parenchyma.

Investigations revealed a normal electrocardiogram (ECG) and negative troponin levels, measured at 0.0001 ng/mL. Lipasemia levels were recorded at 21.7 IU/L (reference range: 8-78), while hyperleukocytosis was noted at 12,200/mm^3^. Hemoglobin concentration was 14.5 g/dL, accompanied by a platelet count of 422 × 10^3^/L. Both creatinine and blood urea nitrogen values fell within acceptable ranges. Lipid profiles exhibited moderate disturbances, notably with suboptimal levels of high-density lipoprotein at 0.37 g/L, whereas serology for *Helicobacter pylori* yielded negative results. This moderate hyperleukocytosis could potentially be linked to a bronchopulmonary infection.

Through clinical-biological investigation, the presence of myocardial infarction in the inferior region was ruled out by ECG results and troponin levels. The primary concern was the potential occurrence of a small bowel volvulus, a condition frequently unveiled through the identification of this anomaly. Our therapeutic approach included administering symptomatic medication and observing the patient to prevent complications.

## Discussion

Intestinal malrotation is a congenital developmental anomaly. It is a rare entity, occurring in 1 in 6,000 cases, with 90% of cases diagnosed in the first year of life [2]. Recent studies have reported a higher incidence of 1 in 500 births [8]. The increase in incidence may demonstrate the importance of advances in imaging detection, although the validity of these incidences remains questionable. The incidence of this anomaly in adults is estimated between 0.00001% and 0.19% [1], and autopsy diagnoses have revealed population figures of around 0.03% [9]. This anomaly is frequently associated with congenital malformations and morbidity, making it essential to evoke this diagnosis at an early stage by a CT scan with contrast injection to optimize patient management [1,5].

In adults, the symptoms of malrotation are nonspecific and multifaceted [2,4]. Dominated by abdominal pain, with 76.8% in a literature review by Neville et al., constipation, nausea, vomiting, and bowel obstruction may be observed [10]. Rarely, pancreatic symptoms are described in the literature, with a prevalence of 2.1% [6,10]. The abnormal anatomical relationship between the duodenum and pancreas may be responsible for intermittent obstruction of the pancreatic duct [11].

The physiopathology of malrotation has not been identified, but some authors such as Martin et al. have identified a genetic factor, demonstrated by the association of malrotation with mutations in the forkhead box transcription factor and L-R asymmetry genes [12]. Other factors include consanguinity, recessive or dominant transmission, chromosomal imbalance, and environmental factors. In other conditions, intestinal malrotation is a syndrome of an associated underlying pathology, such as Martinez-Frias syndrome [5,12].

The embryogenesis of the primitive intestine, described by Meckel in 1817 and later elucidated by Mall in 1898, involves three key stages of rotation. Initially positioned outside the abdominal cavity, the primitive loop undergoes sequential counterclockwise rotations, ultimately aligning the duodenojejunal angle to the right of the superior mesenteric artery (SMA) and the ileocaecal junction to its left [7,13]. By the 10th week, a second rotation integrates the primitive loop into the abdominal cavity, positioning the ileocecal junction in the subhepatic region. The third stage, occurring at 11-12 weeks, completes a 270° rotation, establishing the final configuration with the duodenojejunal angle below the SMA and the ileocecal junction in the right flank [4,7]. This allows the root of the mesentery to be lengthened. In this way, modal anatomy is achieved. Abnormal rotation represents a premature cessation of the embryonic rotation process at various stages. Stringer introduced a classification system based on the developmental stage of the rotation error. This classification comprises the following three types: type 1 (no rotation), type 2 (duodenal malrotation), and type 3 (combined duodenal and caecal malrotation) [14].

Congenital anomalies are very common in intestinal malrotation, representing up to 60% [5]. Therefore, an intestinal rotation anomaly during embryogenesis implicates the duodenum and disrupts the development of the pancreas [6,11]. The majority of the literature describes a hypoplastic or aplastic defect in the process of unciform development [6,11]. Chandra et al. reported 86% abnormal development of the pancreatic head in a series of 25 cases with nonrotational intestinal, of which 86% had aplasia or hypoplasia [11]. This malformation may be associated with an anomaly of the pancreatic ducts, exposing patients to the risk of plication of the Wirsung and causing a defect in the draining of the pancreas [6].

The inversion of the ratio of the superior mesenteric vessels, in which the artery passes to the right and behind the vein, is another essential feature described in many studies [2,15]. Vessel orientation is an essential element observed in cross-sectional imaging but is not diagnostic [10]. Chandra et al., in a series of 25 cases, found 90% of cases with this anomalous position of the mesenteric vessels [11]. In a systematic review of 194 cases, Neville et al. found 58% inversion of the SMA/SMV ratio [10]. The imaging modality serves as an exemplary diagnostic tool, and contrast-enhanced CT continues to retain its status as the gold standard [9], boasting a sensitivity rate of 97.5%, as reported in the systematic review conducted by Neville et al. [8,10]. A meticulous evaluation encompassing the entirety of the intestine, coupled with a discerning differentiation between the superior mesenteric artery and vein, not only informs the diagnostic process but also enhances our comprehension of the diagnostic findings.

This nonrotation (type 1) case included a left-positioned cecum and ascending colon with a duodenojejunal junction to the right of the rachis, accompanied by an inversion position of the superior mesenteric vessels and aplasia of the uncinate process of the pancreas.

The essential role of imaging, especially CT scans, is particularly emphasized in this context, and this work encourages radiologists to be extremely familiar with this pathology to predict the ultimate complications, which remain small bowel volvulus or small bowel obstruction.

## Conclusions

This case highlights a rare presentation of malrotation in an adult with aplasia of the acinar process of the pancreas, which manifested as acute abdominal pain, raising suspicion of pancreatitis. It is rarely described, in the literature that intestinal malrotation can manifest as pain suggesting pancreatitis, and many studies have described that this congenital anomaly is discovered incidentally during surgery for conditions other than intestinal malrotation, such as appendectomy.

However, these patients have previously benefited from radiological examinations such as CT scans and are sometimes referred back to surgery. Therefore, we emphasize the importance of raising awareness among practitioners, especially radiologists, as early detection and accurate radiological assessment of this pathology play an essential role in diagnosing, guiding appropriate therapeutic strategies, and ensuring that potential complications associated with intestinal malrotation are not missed.
